# The influence of experimental infection of gilts with swine H1N2 influenza A virus during the second month of gestation on the course of pregnancy, reproduction parameters and clinical status

**DOI:** 10.1186/1746-6148-10-123

**Published:** 2014-06-04

**Authors:** Krzysztof Kwit, Małgorzata Pomorska-Mól, Iwona Markowska-Daniel

**Affiliations:** 1 Department of Swine Diseases, National Veterinary Research Institute, Partyzantów 57, 24-100 Pulawy, Poland

**Keywords:** H1N2 influenza A virus, Experimental infection, Reproduction disorders, Gilts

## Abstract

**Background:**

The course of swine influenza in pigs is reported to be similar to human influenza. Occasionally abortions and other reproduction disorders have been associated with influenza A virus (IAV) infection in pigs. Abortions may be a consequence of high fever, pro-inflammatory cytokines or transplacental transmission of the virus.

The role of IAV in the complications observed during pregnancy has been scanty and the true importance of this agent as a cause of reproductive problems in swine is not known. The aim was to determine the possible involvement of swine H1N2 IAV strain on reproductive disorders in pregnant gilts under experimental conditions.

**Results:**

The gestation length was from 113 to 116 days, no abortion or any other reproduction disorders were noted. A PCR assay confirms the presence of IAV in the nasal swabs taken from inoculated gilts between 1 and 5 dpi. In the nasal swabs from control gilts and newborn piglets, no IAV genetic material was found. No viral RNA was detected in samples of blood taken from gilts and piglets, placentas, lungs and tracheas taken from piglets euthanized after delivery. The significant decrease in the number and percentage of lymphocytes without leukopenia was observed at 4 dpi in inoculated gilts. The percentage of granulocytes increased significantly at 4 dpi in inoculated pigs. The concentration of IL-6, IL-10 and TNF-α were higher in inoculated gilts, while IL-4 and IFN-γ were not detected in the serum of any of animals. The serum concentrations of C-reactive protein remained stable during study, while haptoglobin concentrations increased significantly after inoculation.

**Conclusions:**

The results of the study indicate that infection of pregnant gilts with swine H1N2 IAV in the second month of pregnancy does not cause abortion and other reproduction disorders. No evidence for transplacental transmission of swine H1N2 IAV was found. However, due to subclinical course of influenza in the present experiment caution should be taken in extrapolating these results to the cases of acute influenza. The other limitation is IAV diversity. It cannot be excluded that other subtypes of IAV could be associated to reproduction failure in pigs.

## Background

Swine influenza is characterized by high morbidity, low mortality and serious economic consequences [1]. In pigs, the disease is reported to be very similar to human influenza: high fever, lethargy, coughing and labored breathing, anorexia and weight loss [1,2]. Sneezing, conjunctivitis, nasal discharge may also be observed [1,2]. Occasionally abortions, fetal deaths and other reproduction disorders have also been associated with IAV infection in pregnant pigs [3-8].

Although in human certain infections are well recognized to increase the risk for adverse pregnancy outcomes, the effects of maternal influenza infection on the fetus are not well understood. In pregnant sows, the influence of IAV on the course of pregnancy as well as on fetus is still poorly known. Most previous studies have been performed during 50s-80s of the last century [3-5]. Studies with IAV in pigs conducted in the USA indicated that pigs born alive from dams inoculated during the first stages of gestation with live IAV had higher mortality rates and lower weaning weights than those from control dams [9]. Moreover, sporadic abortions late in pregnancy and increased stillbirths have also been reported during swine influenza outbreaks in Europe and in North America [6,10,11]. In the study described by Madec et al. [5] only 3 of 13 sows in their 1st week of pregnancy completed gestation and farrowed pigs, in 2 of 8 sows that were 1 month into gestation total embryonic resorption occurred, and in the 18 sows that were more than 45 days into gestation a single abortion occurred [5]. The reproductive disorders related to influenza A virus infection in pigs were also reported previously by Pejsak and Markowska-Daniel [12].

Abortions in IAV infected individuals (humans, pigs) may be a consequence of high fever and pro-inflammatory cytokines as well as transplacental transmission of the virus, what was confirmed for other influenza viruses [5,13]. Both animal and human epidemiologic studies suggest that hyperthermia is often associated with an increased risk for adverse outcomes [14,15]. Factors that might limit this risk include shorter fever duration and use of fever-reducing medications [16].

Viremia is believed to occur infrequently in human influenza and to date was not confirmed for pigs [17]. Transplacental transmission of the virus also appears to be rare [4,5,13]. However, even in the absence of transmission through the placenta, the influenza virus may have an adverse effect on the fetus, what was confirmed for both animals and humans [13,14,18].

The role of IAV in the complications observed during pregnancy in pigs has been scanty and the true importance of this agent as a cause of reproductive problems in swine is not known. So far, the pathogenic mechanisms involved in the disturbances in reproduction in the course of influenza have not been finally clarified. The following study was planned to define the effect of swine H1N2 IAV infection on the course of pregnancy and porcine fetus. Influenza A virus (H1N2 subtype) has been isolated previously from farms with reproductive disorders but the role of IAV on swine reproduction is not clear [6,19]. The main objective of this study was to determine the effect of an H1N2 IAV on reproductive disorders in pregnant gilts under experimental conditions.

## Results

### Clinical signs and pathological examination

No significant clinical signs were observed in pregnant gilts inoculated intranasally (IN) and intratracheally (IT) with IAV H1N2. Control, mock-inoculated gilts also did not develop any signs of the disease. The rectal temperatures were below 40°C. Individual rectal temperature in inoculated and control gilts ranged from 38.5-39.3°C. Clinical score for all gilts was equal 0. Postmortem examination of euthanized piglets did not reveal typical lesions deriving from IAV infection.

### Reproduction and production parameters

The gestation length was from 113 to 116 days, no abortion or any other reproduction disorders were noted. No clinical signs characteristics for IAV infection in pigs were observed in any of the piglets born from infected and control gilts. The average production and reproduction parameters for all experimental groups are presented in Table 1.

**Table 1:** Reproductive and production parameters of gilts inoculated with swine H1N2 influenza A virus compared to control gilts (mean ± SD)

	**Average gestation length**	**Average pigs/litter**	**Average pigs born alive/litter**	**Stillborn pigs/litter**	**Mummified pigs born/litter**	**First week mortality/litter**	**Average liter weight (kg)**	**Average birth weight (kg)**
Gilts inoculated intranasally	115.0 ± 0.70	12.33 ± 0.58	12.33 ± 0.58	0 ± 0.00	0 ± 0.00	0.60 ± 0.89	17.38 ± 2.70	1.37 ± 0.08
Gilts inoculated intratracheally	114.0 ± 0.54	12.00 ± 2.65	12.00 ± 2.65	0 ± 0.00	0 ± 0.00	0.80 ± 0.83	16.38 ± 2.63	1.38 ± 0.09
Control gilts	114.6 ± 0.54	12.33 ± 1.52	12.33 ± 1.52	0 ± 0.00	0 ± 0.00	0.60 ± 0.54	17.16 ± 0.90	1.40 ± 0.10

### Hematological examination

The results of the present study demonstrated that in H1N2 IAV inoculated gilts, the overall number of leukocytes remained stable during study and ranged from 16.46 × 10^9^/l to 24.12 × 10^9^/l. In control gilts the overall number of leukocytes ranged from 16.23 × 10^9^/l to 23.15 × 10^9^/l. No significant differences were found between both inoculated groups and control gilts (p > 0.05). There was a significant decrease in the number and percentage of lymphocytes in gilts at 4 and 7 days post inoculation (dpi) respectively compared to control gilts (p < 0.05). The percentage of granulocytes increased significantly at 4 dpi in inoculated pigs (p < 0.05). The mean hematological parameters (±SD) in gilts inoculated with swine H1N2 IAV and in control gilts (relative and absolute numbers) are presented in Table 2.

**Table 2:** The mean hematological parameters (±SD) in gilts inoculated swine H1N2 influenza A virus and in control gilts (relative and absolute numbers)

	**Control**	**IT inoculated**	**IN inoculated**
**0 dpi**			
WBC × 10^9^/l	20.22 ± 4.09	22.13 ± 2.92	19.72 ± 4.66
LYM × 10^9^/l	11.64 ± 3.01	12.06 ± 4.29	10.39 ± 4.20
GRA × 10^9^/l	8.35 ± 1.86	9.90 ± 1.36	9.19 ± 1.57
LYM (%)	57.45 ± 6.76	53.70 ± 12.31	51.54 ± 9.48
GRA (%)	41.43 ± 5.98	45.55 ± 12.18	47.76 ± 9.42
**4 dpi**			
WBC × 10^9^/l	17.21 ± 5.91	19.49 ± 4.45	17.99 ± 5.35
LYM × 10^9^/l	8.33 ± 2.68	4.85 ± 2.47*	5.04 ± 1.78*
GRA × 10^9^/l	9.37 ± 6.50	13.72 ± 3.06	12.30 ± 4.72
LYM (%)	48.40 ± 8.45	22.90 ± 5.05*	29.17 ± 8.95*
GRA (%)	54.44 ± 8.58	71.90 ± 5.70*	67.08 ± 8.36*
**7 dpi**			
WBC × 10^9^/l	20.09 ± 4.81	21.40 ± 2.95	19.40 ± 4.05
LYM × 10^9^/l	10.75 ± 5.38	8.91 ± 8.95	8.28 ± 6.43
GRA × 10^9^/l	9.35 ± 0.91	11.46 ± 4.76	10.39 ± 3.85
LYM (%)	50.07 ± 7.41	39.13 ± 3.48*	41.24 ± 7.05
GRA (%)	49.97 ± 7.46	55.61 ± 9.93	54.91 ± 6.20
**14 dpi**			
WBC × 10^9^/l	17.11 ± 0.94	17.06 ± 1.52	17.89 ± 1.32
LYM × 10^9^/l	8.06 ± 1.61	7.70 ± 4.40	9.11 ± 1.75
GRA × 10^9^/l	8.95 ± 2.43	9.26 ± 1.13	8.67 ± 2.09
LYM (%)	47.10 ± 4.56	45.13 ± 1.51	50.92 ± 10.71
GRA (%)	52.30 ± 4.43	54.27 ± 8.68	48.46 ± 10.49
**28 dpi**			
WBC × 10^9^/l	18.30 ± 0.59	19.01 ± 0.18	18.82 ± 0.79
LYM × 10^9^/l	9.05 ± 1.88	9.51 ± 0.74	9.42 ± 0.51
GRA × 10^9^/l	8.70 ± 1.39	9.41 ± 0.91	8.96 ± 0.78
LYM (%)	49.45 ± 8.42	50.02 ± 5.41	50.05 ± 3.32
GRA (%)	47.54 ± 9.46	49.50 ± 5.40	47.60 ± 3.57

WBC – white blood cells, LYM- lymphocytes, GRA-granulocytes.

*significant compared to control group (p < 0.05).

### In vitro cellular response

**Figure 1:**
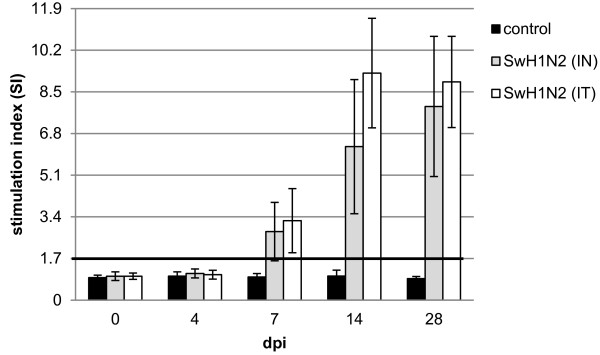
The mean SI values (±SD) calculated for gilts infected intranasally (IN) or intratracheally (IT) with swine H1N2 influenza A virus and for control gilts.

The mean values of stimulation index (SIx) in control pigs and pigs from experimental group before inoculation ranged from 0.78 to 1.15 (in this study the SIx > 1.69 indicates antigen-specific proliferation, see Material and Method for details). Seven days after inoculation all intratracheally inoculated gilts developed an antigen-specific proliferation (an individual SIx higher than 1.7), while in 1 out of 5 IN inoculated gilts no antigen-specific proliferation was found at that time. The mean SIx values (±SD) in inoculated and control gilts are presented in Figure 1.

### Antibody response against IAV H1N2

All inoculated gilts exhibited specific antibodies against hemagglutinin (anti-HA) in the serum from 14 dpi to the end of study. The highest HI titres were observed at 14 dpi. In gilts infected IN the HI titres were generally lower (144.00 ± 35.77 and 88.00 ± 43.81, respectively for 14 and 28 dpi) than in IT infected gilts (176 ± 87.63 and 104 ± 53.66, respectively for 14 and 28 dpi). The uninfected gilts had no detectable HI serum antibodies. No antibodies against HA were detected in the serum taken from colostrum free piglets.

### Molecular examination of swabs and tissues

A RRT-PCR assay which was used to confirm the presence of IAV in the nasal swabs, revealed positive results from inoculated pigs between 1 and 5 dpi (Table 3).

**Table 3:** Real time RT-PCR results for clinical samples (nasal swabs) from gilts inoculated with swine H1N2 influenza A virus and control gilts

		**Times post-infection (days)**					
**Gilts**		**0**	**1**	**2**	**3**	**4**	**5**
IN inoculated	1	-	++	++	++	++	++
	2	-	++	++	++	++	+
	3	-	-	+	++	++	+
	4	-	+	++	++	++	+
	5	-	-	+	++	++	+
IT inoculated	1	-	+	++	++	++	++
	2	-	++	++	++	++	+
	3	-	+	++	++	++	++
	4	-	-	+	++	++	++
	5	-	++	++	++	++	+
Controls	n = 5	-	-	-	-	-	-

Real-time RT-PCR results are given as ++ (Ct value <30; positive), + (Ct value 30-35; weak positive), - (Ct value >35, negative), – negative.

No IAV was detected by RRT-PCR in the nasal swabs taken before inoculation, nasal swabs taken from control gilts and newborn piglets, samples of blood taken from gilts and piglets, samples of lungs and tracheas taken from piglets euthanized after delivery and placentas.

### Systemic cytokine response

**Figure 2:**
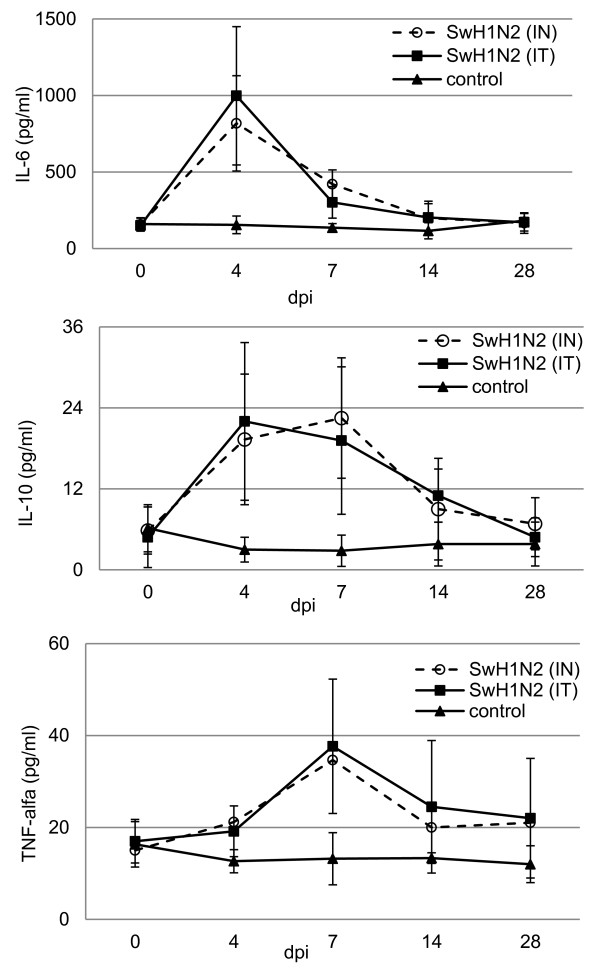
The mean cytokines concentration in serum of gilts infected intranasally (IN) or intratracheally (IT) with swine H1N2 influenza A virus and control gilts.

Concentrations of IL-4, IL-6, IL-10, IFN-γ and TNF-α in the serum of gilts were evaluated on 0, 4, 7, 14 and 28 dpi. In general the concentration of IL-6, IL-10 and TNF-α were significantly higher in the serum of the inoculated gilts, while IL-4 and IFN-γ were not detected in the serum of any of animals. The mean serum concentration of all investigated cytokines is shown in Figure 2.

The mean serum concentration of IL-10 were significantly higher in inoculated gilts from 4 to 7 dpi (p < 0.05). There were no significant differences between both inoculated groups of gilts. At 4 dpi, also the mean serum concentration of IL-6 was significantly higher in inoculated gilts from both groups, compared to controls (p < 0.05). With regard to TNF-α the significantly higher concentration, compared to control, was observed only at 7 dpi (p < 0.05) in both IN and IT infected gilts. Starting from 10 dpi the concentrations of IL-6, IL-10 and TNF-α in serum of inoculated gilts did not differ significantly from those observed in control gilts (p ≥0.05).

### Acute phase proteins response

**Figure 3:**
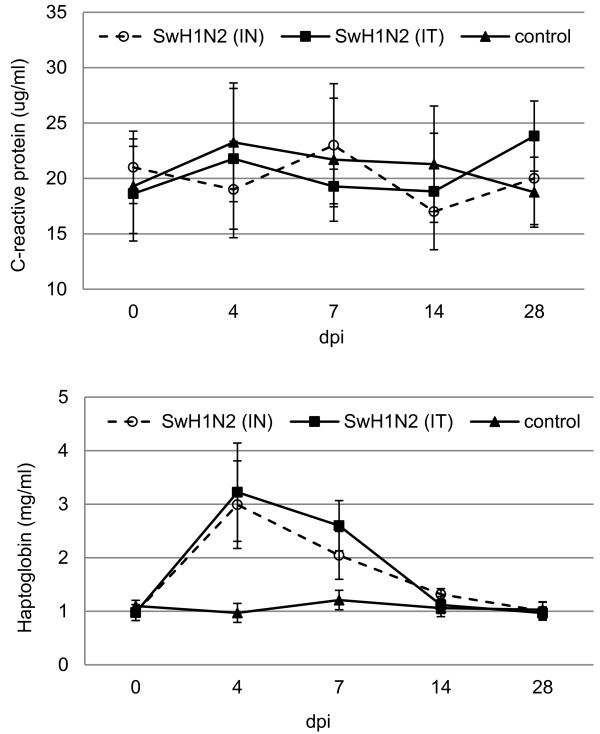
The mean concentrations of CRP and Hp in serum of gilt before and after intranasal (IN) or intratracheal (IT) inoculation with swine H1N2 influenza A virus, and in the control groups.

The time course of mean C-reactive protein (CRP) and haptoglobin (Hp) concentrations (±SD) in inoculated and control gilts during the study period is presented in Figure 3.

The concentrations of CRP remained stable after inoculation, and ranged from 17.05 to 23.25 μg/ml. No significant difference was found between CRP concentration in serum of inoculated and control gilts (p ≥ 0.05). In contrast, Hp concentrations increased significantly after inoculation in gilts from both infected groups, with mean maximum levels observed at 4 dpi (p < 0.05). Preinoculation individual level of Hp were found to be below 1.06 mg/ml, and the highest individual induced level reached 4.1 mg/ml in IT infected gilts and 3.95 mg/ml in IN infected gilts. Significantly higher concentration of Hp in inoculated gilts were also observed at 7 dpi (p < 0.05). Changes in serum Hp concentrations were observed in all swine H1N2 IAV infected gilts. The mean peaked level was over 3-fold higher compared to the mean preinoculation concentration. From 14 dpi the CRP and Hp concentrations had decreased and did not differ significantly between control and infected gilts (p ≥ 0.05).

## Discussion

Reproductive failure in pigs can be caused by a variety of infectious and non-infectious factors, including poor environmental conditions and poor hygiene. Among different infectious agents causing reproductive disorders in pigs, viral infections of pregnant gilts and sows result in the biggest economic losses [8,12]. According to the results obtained previously [5,6,10,11] the influenza virus, in addition to respiratory symptoms can cause abortions and other reproduction disorders in both humans and animals, including pigs. Abortions observed during influenza infection may be a result of high fever and proinflammatory cytokines as well as transplacental transmission of the virus [4,5,13]. The knowledge about the possibility of intrauterine infection of fetus with influenza virus is limited. Infection with influenza virus in pregnant women, especially during the first trimester of pregnancy, can lead to intrauterine infection of the embryo, which was confirmed by virus isolation from both the placenta and the aborted fetal tissue [13,20,21]. However, because of different type of placenta these results cannot be simply transferred to pigs. With regard to pigs there are only partial data from the field observations on the occurrence of abortion as a result of IAV infection of gestating sows [4,5,9-12,22]. The results published previously indicate that the influenza related reproduction disorders may vary depending on the gestation period in which the infection occurred [4,5,9-11,22]. The possibility of transplacental transmission of IAV in the course of influenza in pregnant sows was evidenced for the first time by Menšík et al. [3] and confirmed later by others [4,5].

It the present study the naïve gilts from the herd with no history of influenza were used In order to confirm the status of gilts with regard to influenza A virus before the experiment they were checked by PCR (nasal swabs) and HI assay (antibody). Hemagglutination inhibition is a gold standard for detection of antibodies against influenza A virus recommended by the WHO and OIE [23,24]. It should be mentioned that the gilts were not tested for the presence of antibodies against nucleoprotein (NP) of IAV (which is highly conserved among IAV). Previous expose to other IAV may not prevent infection but it may decrease virus replication, and in this way it may be associated with reduced clinical signs. However, because the IAV that currently circulate among pigs in Europe were used in the HI assay, the probability of false negative results is rather low.

In our study no reproduction disorders were observed in pregnant gilts infected with swine H1N2 IAV during second month of gestation. There were no significant differences between inoculated and control sows with regard to all investigated production and reproduction parameters. Moreover in our study no evidence for transplacental transmission of IAV was found. In samples taken from piglets (nasal swabs, blood, tracheas and lungs) as well as in placentas no SIV genetic material was found. All colostrum free piglets were negative for HA specific antibodies. These findings are in agreement with the results of our previous study [22] in which the influence of IAV infection during first month of gestation on the course of pregnancy and reproduction parameters were investigated. In contrast, in a study conducted by Wallace and Elm [4] there was evidence of transplacental transmission of virus in one of ten piglets born to pregnant gilts that were exposed to the H1N1 IAV 10, 24, and 39 days before parturition and a large number of (2-3 per litter) dead piglets were born by infected gilts. Different results can be consequence of using of other subtype of IAV in a Wallace and Elm [4] study. In relation to the great IAV diversity, it is not possible to rule out that different IAVs can have other effects on the course of pregnancy and disturbances in the reproduction.

In the study performed by Wesley [7] with the use of H3N2 IAV all infected females showed no clinical symptoms of infection, what is in agreement with our results. However, in contrast to our investigations, Wesley [7] used seropositive pigs in his study. For confirmation of IAV infection the molecular examination of nasal swabs, HI assay and lymphocyte proliferation tests were performed in the present study. In all inoculated gilts the seroconversion and antigen-specific proliferation were found. Additionally, all inoculated gilts became actively infected and shed IAV between 1 and 5 dpi.

The hematological investigations showed a significant drop in lymphocyte numbers and percentage with no accompanying leucopenia, in inoculated gilts. There was over 50% drop in mean number of lymphocytes between 0 and 4 dpi, while the total number of white blood cells remained unchanged. In IT inoculated gilts the significantly lower percentage of lymphocytes was also observed at 7 dpi. Additionally, a rise over 30% in the mean percentage of granulocytes was observed between 0 and 4 dpi. Previously, in adult human patients with influenza A, relative lymphopenia was found to be an early and reliable laboratory finding for influenza infection [25,26]. Peripheral lymphopenia was also reported in humans infected with highly pathogenic avian influenza H5N1 [27]. The induction of lymphocyte apoptosis by death receptor ligands may play a role in lymphopenia related with influenza infection [28]. In accordance with our findings, relative lymphopenia without leucopenia was previously found in human with positive test results for H1N1. In the field of human medicine, it was suggested [29] that relative lymphopenia might be a marker for H1N1 and thus could also be used to prioritize H1N1 PCR testing if the emergency department’s ability was exceeded [29].

In the present study the concentration of Hp, IL-6, IL-10 and TNF-α were significantly higher in serum of the inoculated gilts compared to control, while IL-4 and IFN-γ were not detected in the serum of any of animals. It should be noted that the respiratory tract is the major site of influenza virus replication suggesting that this is the initial site of cytokine production with a subsequent ‘spill-over’ into the circulation. It is therefore likely that serum levels of these mediators are probably significantly lower than BALF or lung levels. TNF-α is a cytokine of innate immunity. The main effects of TNF-α activity include neutrophil activation, fever, synthesis of acute phase proteins and apoptosis of cells. According to Damjanovic et al. [30] TNF-α is not essential for influenza clearance however, this cytokine is critically required for negatively regulating the extent of lung immunopathology during acute influenza infection. In our study we found significantly higher concentration of TNF-α in the serum of infected gilts only at 4 dpi. The significant increase in the serum concentration of TNF-α and IL-6 during influenza was also reported by Hayden et al. [31] in human patients. IL-6 is a cytokine of innate immunity and regulates inflammation and transition from innate to adaptive immune responses. In present study, in accordance with the results obtained by Hayden et al. [31], the concentration of IL-6 increased during early stage of infection. Lauder et al. [32] reported that IL-6 has an essential role in orchestrating anti-influenza immunity through an ability to limit inflammation, promote protective adaptive immunity and prevent fatal immunopathology. Furthermore, recent data using a mouse model of influenza infection indicate that mice lacking IL-6 are less likely to survive the infection thereby implying a beneficial effect of IL-6 in controlling the infection [32]. The early secretion of this cytokine may therefore constitute an important line of defense against a fatal course of influenza.

In present study the significantly higher level of IL-10 as compared to controls, were detected from 4 to 7 dpi. IL-10 is a cytokine with pleiotropic effects in immunoregulation and inflammation, however it plays mainly an anti-inflammatory role in the immune system. The role of IL-10 during acute influenza virus infection appears to be contradictory. Sun et al. [33] previously found that inhibition of IL-10 signaling during an ongoing influenza resulted in increased inflammation and decreased survival, whereas McKinstry et al. [34] reported that inhibition of IL-10 signaling before infection enhanced viral clearance and increased survival. It seems that the role of IL-10 during influenza is probably dependent on the timing of IL-10 signaling. Additionally, a balance in the levels of pro- and anti-inflammatory cytokines may be crucial in host defense against influenza virus infection [35].

The acute phase proteins and cytokines associated with SI in pigs were also investigated by Barbé et al. [36] during first 5 dpi after inoculation of pigs with H1N1 strain of IAV. In general the serum concentrations of CRP and Hp peaked at 2 dpi, but no significant differences were found between infected and control pigs. However, it could be a result of small number of pigs (3 per group) under study of Barbé et al. [36]. The finding of IFN-γ in serum in a Barbé et al. [36] study, contrasts with results of present study and the results of previous investigations [22,37]. After infection of pigs with a North American H3N2 isolate of IAV no change was found in the serum IFN-γ concentration [37]. Factors that may have attributed to the greater, more rapid IFN-γ response in the study of Barbé et al. [36] may be the larger inoculation dose used and the use of young 3-week-old caesarean-derived, colostrum-deprived pigs, which resulted in more severe course of infection.

Increased plasma concentrations of various cytokines have also been reported in, hospitalized human patients with H1N1 [38] and highly pathogenic avian H5N1 infection [39]. In these patients, plasma cytokine concentrations tightly correlated with viral RNA levels in nasopharyngeal or throat swabs, as well as with clinical severity ‘scores’. However in mentioned studies the severe, clinical course of influenza was observed in contrast to our results.

The acute phase response in pigs after infection with various subtypes of IAV, including H1N2, was also investigated by us previously [40,41]. Similarly to the results of the present study the significant increase of Hp, but no CRP serum concentration were found in pigs with subclinical influenza [41]. In contrast during acute influenza the concentration of CRP increased significantly [40].

## Conclusion

Summarising, the results of the present study indicate that infection of pregnant gilts with swine H1N2 IAV in the second month of pregnancy does not cause abortion and other reproduction disorders. No evidence for transplacental transmission of swine H1N2 IAV was found. However, because of different type of placenta, these results cannot be reliably extrapolated to others species, including humans. Due to subclinical course of influenza in the present experiment caution should be taken when extrapolating these results to the cases of acute influenza. The other limitation of the present study is IAV diversity. It cannot be excluded that other subtypes of IAV could be associated to reproduction failure in pigs.

## Methods

### Animals

A total of 15 gilts, France hybrids FH 900 at the age of approximately 8 months and their piglets were used in the study. Pigs were randomly allocated into 3 groups. Ten gilts were infected with H1N2 IAV (intranasally (IN) n = 5, or intratracheally (IT) n = 5), following 5 served as controls. Sample size calculations were performed in STATISTICA 8.0 (StatSoft). All gilts prior to the start of the study were shown to be both influenza A virus and antibody (subtypes H1N1 (A/Swine/Cotes d’Armor/0388/2009, GenBank accession number FR871194), H1N2 (A/swine/Granstedt/IDT3475/2004, GenBank accession numbers: GQ161164.1, GQ161166.1, GQ161165.1, GQ161160.1, GQ161163.1, GQ161162.1), H3N2 (A/swine/Flanders/1/1998; GenBank accession number FJ842116), pandemic H1N1 2009 (A/California/04/09, GenBank accession number: not available) negative by Matrix (M) gene real time RT-PCR and haemagglutination inhibition assay (HI), respectively. Additionally gilts were seronegative against PRRSV, pseudorabies virus and leptospiras. All sexually mature gilts were inseminated. The pregnancy was confirmed with the use of ultrasonography at 24 and 58 days post insemination. Complete management and health data for the gilts and their offspring were maintained. The prophylactic program at the time of pregnancy consists of vaccination of the gilts with inactivated vaccines against parvovirus infection, erysipelas and colibacillosis*.* During the experiment, pigs were housed in isolated units, one for the control gilts and one for each group of infected gilts. The condition of piglets was monitored to weaning. Animal use and handling protocols were approved by Local Ethical Commission (University of Life Sciences in Lublin, Poland).

### Preparation of virus inoculum

Swine influenza virus A/sw/Granstedt/IDT3475/2004 (subtype H1N2) (hereafter referred to as H1N2 IAV), kindly provided by IDT (Germany) was used for the experimental infection. The virus concentration was evaluated in Madin-Darby canine kidney (MDCK) cells and stored at -80°C until used. Virus titers were calculated by the Reed-Muench method.

### Experimental design

Fifty eight days post insemination (day 0), ten gilts were inoculated with H1N2 IAV. Inoculations of 3 × 10^7.3^ TCID_50_ of virus in 5 ml of phosphate-buffered saline (PBS) were given IN to each nostril (total infection dose 6 × 10^7.3^ TCID_50_) or IT (needle method; total infection dose 6 × 10^7.3^ TCID_50_). Five mock-inoculated pigs (with 9.7 ml of PBS and 0.3 ml of allantoids fluid) served as control pigs.

Rectal temperatures were assessed and clinical signs of disease were recorded daily during first 7 days post inoculation (dpi), however, the general condition and health status of gilts was monitored to the end of pregnancy. After inoculations, gilts were observed and scored for the respiratory signs as follows: respiratory rate: 0- normal, 1 – slightly elevated, 2 – moderately elevated, slight abdominal breathing, 3 – clearly elevated, distinct abdominal breathing; nasal discharge 0 – absent, 1 present; coughing 0 – absent, 1 present; sneezing 0 – absent, 1 present. All scores per topic are accumulated for a total clinical score of each individual pig (0-6). Rectal temperature was also measured daily. Fever was recorded when the rectal temperature ≥ 40°C.

Blood samples from gilts were collected on days -7, 0 (inoculation), 4, 7, 14 and 28 dpi. The blood was also taken from piglets before consuming colostrum (colostrum free piglets, 5 piglets per litter). Serum was harvested after centrifugation and stored at -20°C for further analyses.

Nasal samples were taken from gilts at 0, 1, 2, 3, 4 and 5 dpi and from piglets immediately after birth.

The parturitions were monitored by a veterinary surgeon. All placentas were collected at delivery.

Additionally, the weakest piglet from each litter was euthanized and complete necropsy was done, with special emphasis on the respiratory tract. Gross lung lesions were assessed for the presence or absence of pulmonary cranioventral multifocal consolidation and when present, extension was recorded. Samples from lung and tracheas, as well as placentas were collected aseptically and frozen at -80°C until their use for viral RNA extraction.

### Reproductive and production parameters

For each gilt the following reproduction parameters were determined: average gestation length, number of live-born piglets, number of stillborn piglets, number of mummified fetuses. Additionally the selected production parameters, including individual birth body weight of piglets, litter birth weight, number of piglets dying during first week of life, were recorded.

### Laboratory examination

#### Swabs, blood and tissue samples

The general swine influenza A RRT-PCR method was used for detection of IAV in the blood and nasal swabs of gilts, placentas and in the blood, nasal swabs and lung samples from piglets, as described previously [42]. Viral RNA was extracted from nasal swabs and tissues using the QIAamp Viral RNA extraction kit (Qiagen, Valencia, CA). RRT-PCR was performed using a one step RT-PCR kit (Qiagen, Valencia, CA). The oligonucleotides sequences (probe and primers) were specific for matrix gene region of known European swine influenza A viruses, as well as any swine infections that may be due to A (H1N1) pdm-like-2009 influenza A virus. Samples with Ct value <30 were considered to be positive, samples with Ct value 30-35 were considered to be weak positive, samples with Ct value >35 were considered to be negative.

#### Haematological examinations

Whole blood samples were analyzed for different leukocyte proportions and concentrations on a Abacus Junior Vet 5 hematology analyzer (Diatron, Hungary). Proportions of lymphocytes, monocytes and granulocytes were calculated as a percentage of leukocyte concentration.

#### Lymphocyte proliferation assay (LPA)

The T-cell proliferation assay to measure influenza-specific T-cell responses of pigs was performed at 0, 4, 7, 14 and 28 dpi, as described previously [43]. Briefly*,* PBMC were isolated from blood samples by centrifugation onto Histopaque 1.077 (Sigma, USA) and were washed twice with PBS. The isolated PBMC were seeded in plastic vials at a density of 1 × 10^6^ viable cells per vial in 1 ml medium (RPMI 1640 containing 10% fetal bovine serum, 2 mM L-glutamine and 1% of antibiotic-antimycotic solution). For analysis of cellular responses, PBMC were restimulated in vitro with 50 μl of medium containing live H1N2 IAV virus (titer 10^6,8^ TCID_50_/50 μl). In control vials the cells were incubated without the virus (mock-control) or with 5μg/ml of concanavalin A (Con-A) (vitality control). All samples were analysed in triplicate.

After 72 hours of incubation at 37ºC in 5% CO_2_ atmosphere, the cultures were pulsed with 0.5 μCi [^3^H]-thymidine (MP Biomedicals, USA). After 18 hours of incubation, cells were harvested and the incorporated radioactivity was measured in a liquid scintillation counter (Tri-Carb 2500TR, Packard, USA). Proliferation was expressed as stimulation index (SIx) calculated as the number of counts per minute (cpm) for H1N2 IAV stimulated cells divided by the number of cpm for the mock-stimulated cells (in each cases taking mean of triplicate vials).

Based on the SIx values of the control group (mean plus 3 x standard deviation), SI value >1.69 was considered to be positive.

#### Haemaglutination inhibition assay (HI)

Antibodies against IAV were measured using a HI assay. The HI assay was performed according to the standard procedure [44], using 0.5% chicken erythrocytes and 4HA units of strain H1N2 IAV and additionally with the use of other subtypes H1N1 (A/Swine/Cotes d’Armor/0388/2009), H3N2 (A/swine/Flanders/1/1998) and pandemic H1N1 2009 (A/California/04/09). All sera were tested in serial twofold dilutions, starting at 1:20. Samples showing titres of ≥ 20 were considered as positive.

#### Determination of cytokines and acute phase proteins concentration in serum

The ELISA kits specific for porcine IL-4, IL-10, IFN-γ and TNF-α were purchased from Invitrogen Corpotarion (Camarillo, USA). The concentration of IL-6 was determined with the use of IL-6 Pig ELISA Kit from Abcam (Cambridge, UK). The detection limit of kits are 2 pg/ml (IL-4), 45 pg/ml (IL-6), 3 pg/ml (IL-10), 2 pg/ml (IFN-γ) and 3 pg/ml (TNF-α). All assays were performed according to the manufacturers’ protocols. All the samples were run in duplicate.

For determination of C-reactive protein (CRP) and haptoglobin (Hp) commercial ELISAs were used according to the manufacturers’ instructions (Pig C-reactive protein ELISA and Pig haptoglobin ELISA from Life Diagnostics, Inc., USA). For all analyses serum samples were tested in duplicate. Prior to analyses serum samples were diluted as follows: 1:1000 for CRP and 1:35000 for Hp.

The quantity of the cytokines and acute phase proteins was calculated based on standard curve for each protein with the use of FindGraph software program.

#### Statistical analysis

The obtained data were subjected to the W. Shapiro-Wilk’s test of normality and the Levene’a test of equal variances with STATISTICA 8.0 (StatSoft). Nonparametric Kruskal-Wallis tests with post hoc multiple comparisons for comparison of all pairs and Friedman test were used for comparison of means. For all analyses, *P* < 0.05 was considered significant.

## Competing interests

The authors declare that they have no competing interests.

## Authors’ contributions

KK and MPM: performing the experiments (inoculation, necropsy, clinical examination), preparation of the serum and tissue samples, laboratory examination, data collection. MPM: analysis of the experimental data, statistical analysis, interpretation of the results and drafting the manuscript with KK. IMD: Conception of the research idea, designing and supervising the experiment, and manuscript reviewing. All authors have read and approved the final manuscript.
